# Supplementary material for: The interactome of intact mitochondria by cross-linking mass spectrometry provides evidence for coexisting respiratory supercomplexes
Source: Mol Cell Proteomics. 2017 Dec 8;17(2):216–32. doi: 10.1074/mcp.RA117.000470 (PMC5795388; doi:10.1074/mcp.RA117.000470)
Supplement: Supplemental Data [file supp_RA117.000470_133922_0_supp_23978_fzffwf.zip › spectra_annotation/mito_spectra_annotation/430-1-1-1-22-1.pdf]

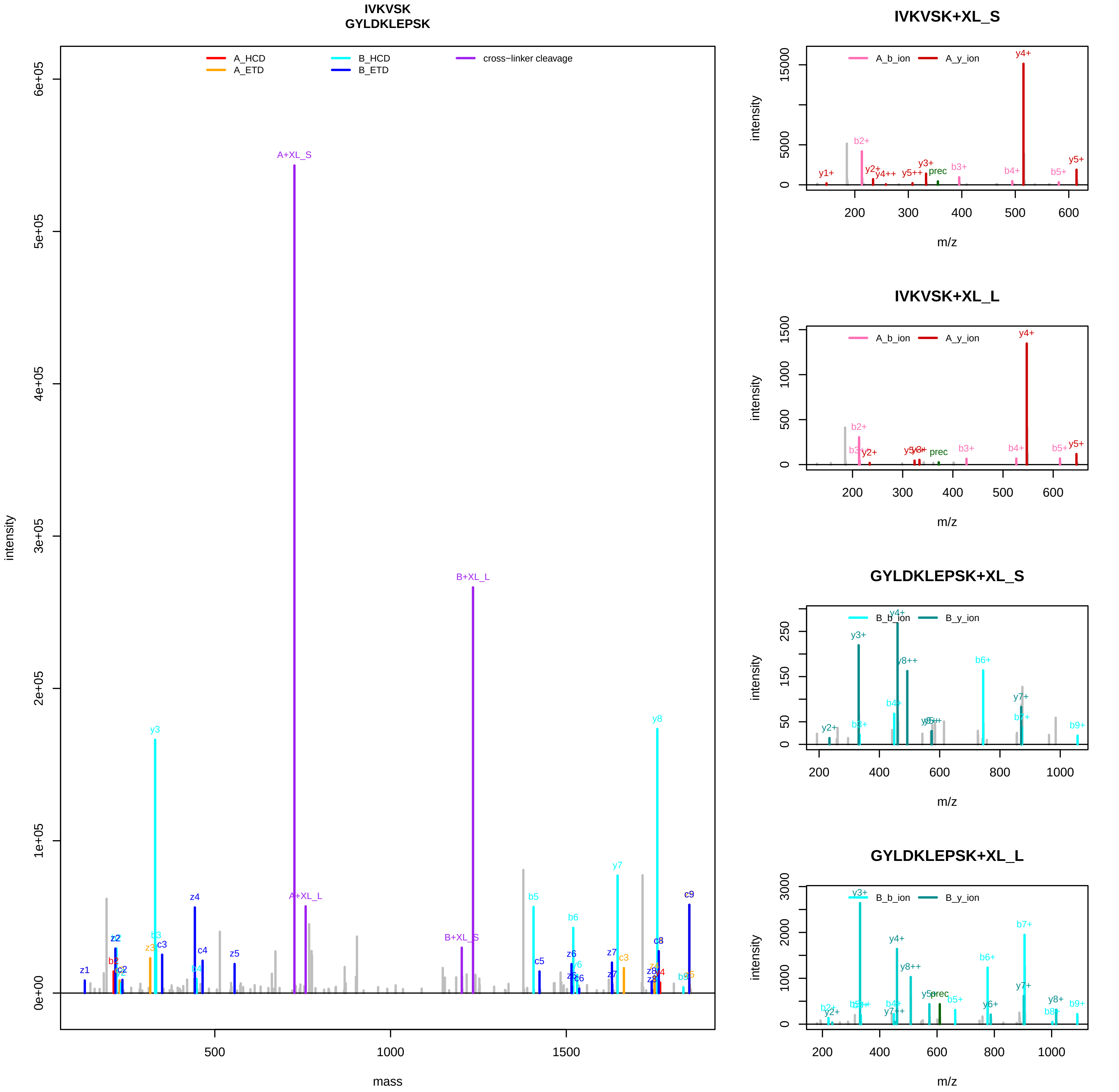

IVKVSK
GYLDKLEPSK
A_HCD
A_ETD
B_HCD
B_ETD
cross−linker cleavage
A+XL_S
B+XL_L
A+XL_L
B+XL_S
y3
y8
y7
b5
b6
c9
c8
z4
c4
z5
c5
z6
z7
c3
b3
z3
b2
z2
z1
intensity
mass
0e+00
1e+05
2e+05
3e+05
4e+05
5e+05
6e+05
500
1000
1500
IVKVSK+XL_S
A_b_ion
A_y_ion
y4+
b2+
y5+
y3+
b3+
b4+
b5+
y2+
y1+
y4++
y5++
prec
intensity
m/z
0
5000
15000
200
300
400
500
600
IVKVSK+XL_L
0
500
1000
1500
GYLDKLEPSK+XL_S
B_b_ion
B_y_ion
y4+
y3+
y8++
b6+
b4+
y7+
b7+
b9+
y2+
b3+
0
50
150
250
200
400
600
800
1000
GYLDKLEPSK+XL_L
y3+
y4+
y8++
b7+
b6+
y7+
y8+
b9+
b8+
y6+
b5+
b4+
y7++
y2+
b2+
0
1000
2000
3000
